# Supplementary material for: The role of selective attention in the positivity offset: Evidence from event related potentials
Source: PLoS One. 2021 Nov 3;16(11):e0258640. doi: 10.1371/journal.pone.0258640 (PMC8565729; doi:10.1371/journal.pone.0258640)
Supplement: S1 Fig — (DOCX) [file pone.0258640.s001.docx]

**S1 Fig. The complete lists of words used as stimuli in the study.**

| **Positive Words** | **Negative Words** | **Neutral Words** |
| --- | --- | --- |
| accept | addict | ankle |
| admire | agony | arms |
| adore | annoy | banner |
| agree | blind | barrel |
| beauty | bloody | bench |
| bless | broken | blank |
| bliss | brutal | board |
| brave | burden | body |
| bright | cancer | bowls |
| care | corpse | butter |
| champ | coward | chair |
| charm | crime | circle |
| cheer | crisis | clock |
| comedy | cruel | corner |
| cuddle | dead | custom |
| cute | detest | doors |
| dazzle | devil | elbow |
| desire | dread | engine |
| devote | drown | fabric |
| enjoy | fail | farm |
| favor | fault | foot |
| free | fear | forks |
| friend | filth | hammer |
| gentle | foul | hands |
| glory | greed | icebox |
| good | grief | iron |
| happy | guilty | kettle |
| honest | hate | knots |
| honor | hurt | lamp |
| hope | idiot | locker |
| humor | injury | market |
| joke | insane | metal |
| jolly | insult | month |
| joyful | invade | name |
| kind | jail | news |
| laugh | liar | paint |
| lively | lonely | paper |
| love | loser | pencil |
| loyal | misery | plant |
| lucky | nasty | rocks |
| merry | pain | ships |
| nice | poison | sphere |
| party | prison | spray |
| peace | rape | square |
| please | regret | statue |
| power | reject | stove |
| pretty | robber | street |
| proud | rotten | table |
| reward | rude | tank |
| safe | shame | taxi |
| secure | sick | tents |
| social | slave | theory |
| strong | stupid | time |
| talent | toxic | tool |
| tender | tragic | tower |
| thank | trauma | trucks |
| treat | ugly | unit |
| trust | vandal | violin |
| truth | victim | wagon |
| useful | vomit | watch |
| virtue | waste | wind |
| wise | whore | window |
