# Supplementary material for: The role of selective attention in the positivity offset: Evidence from event related potentials
Source: PLoS One. 2021 Nov 3;16(11):e0258640. doi: 10.1371/journal.pone.0258640 (PMC8565729; doi:10.1371/journal.pone.0258640)
Supplement: Attachment — Submitted filename: SelectiveAttentionPositivityOffset_PLOS_ResponsetoReviewer.docx [file pone.0258640.s002.docx]

**Response to Reviewers**

Dear Dr. Hinojosa,

We would like to thank you and the reviewer for your consideration of our paper. We have made changes to the manuscript in response to the concerns noted. These are described below, along with additional explanations addressing each of their concerns.

We hope that these revisions, are well received. Changes in the revised manuscript are denoted in the file “SelectiveAttentionPositivityOffset_PLOS_Revised Manuscript with Track Changes” with blue and red font. Page numbers and paragraph information directing reviewers to the revisions noted below refer to the marked up manuscript. Thank you for your time and feedback. We look forward to your response.

Sincerely,

Regard Booy

**Major concerns:**

1. The first major concern the reviewer noted was that word frequency was not controlled for. We agree with the reviewer on the importance of word frequency, and to address this concern we have greatly expanded our discussion of this limitation within the manuscript (see pp. 28 – 29). Additionally, statistical tests of the differences in frequency between the three stimulus categories have been added on p. 13. The tests indicated that there was no significant difference between positive and neutral words (p=.49), but that negative words were significantly less frequent than both positive (p,.01) and neutral words (p=.01). Unfortunately, during the design of the study, it was determined that word frequency differences were unavoidable for practical reasons. When creating the list of words to be included in the study, it was not possible to control for all of the potentially important factors. We needed to take into account valence, arousal, word length, frequency and familiarity. We also needed to have a reasonably even distribution of starting letters since closed and open sounds have emotional connotations. And other semantic connotations further limited the available words. Obviously, words with multiple meanings had to be avoided, and words related to specific phobias could not be included, in order to avoid the possibility of strong personal associations of the words (see Herbert et. al. 2006). It was simply not possible to fully account for all of these variables and we had to prioritize controlling for valence, arousal and word length since these factors were anticipated to be the most likely to affect the ERP components of interest.

There is a further problem of how positive and negative words are used in everyday language. People tend to avoid negative emotions, and this is reflected in language use. Thus, negative words are less frequent than positive and neutral words (Garcia, Garas & Schweitzer, 2012). This problem is exacerbated when trying to control for arousal, since high arousing negative words are especially infrequent. Thus, it proved impossible to build a list of words that controlled for valence, arousal and word-frequency.

Our solution to maintain experimental control over the independent variable was to control for the *behavioural effect* of these extraneous variables by doing the pilot study. By ensuring that there were no systematic differences between participants’ responses to positive, negative and neutral words in terms of reaction time and accuracy, we could be more confident that extraneous variables like frequency were not exerting an undue influence on the results.

Regarding the reviewer’s point that the frequency difference could explain the ERP results, we believe that this is unlikely. The word frequency effects noted by Hauk & Pulvermüller, (2004) occurred between 150-190ms and 320-360ms whereas the LPP time-window in the present study is between 500-700ms post stimulus. Other studies such as Strijkers, Costa & Thierry (2009) also show that word frequency affects earlier ERP components in the 150-200ms range. Thus, it is unlikely that differences in word frequency could account for the late component differences we observed. If word frequency did have an effect on later components, this would most likely have taken the form of a larger N400 to high frequency words (see Grainger, Lopez, Eddy, Dufau & Holcomb, 2012). In the present study, if the results were driven by word frequency, we would expect *a more negative deflection to the higher frequency positive words,* which is the opposite of what we observed. Thus, either the effects of valence is stronger than the effects of word frequency, or more likely, word frequency did not influence the amplitude of the LPP. This discussion has also been added to the limitations section (p. 29)

2. The reviewer’s second concern was regarding the neutral mood state of participants. In the present study, we are certain that participants were in a neutral mood. We did ask participants to rate their mood on a 7-point scale at four different times throughout the task. We then calculated an average mood for each participant. These ratings did not differ significantly from a neutral mood rating of 4. This should have been included in the original manuscript. We apologise for the oversight and thank the reviewer for catching it!

To address the reviewer’s concerns, the mood manipulation check data was added to the methods section of exp. 2 (see p. 20, “Procedure and Design). In short, there was no significant deviation from a baseline neutral mood in the study. We did not measure mood state in exp 1 since the primary purpose was to compare the different versions of the task. We also expanded the explanation of the low arousing conditions in the general introduction as the reviewer requested (p. 5).

**Minor Concerns**

1. As per the reviewer’s request we have expanded the description of the ERP components and the role of selective attention in the introduction (see p. 5 and 6).

Regarding the reviewer’s comments about the EAP component, there is limited research on the component to date. Because it occurs in a similar time window as the EPN and is also elicited by emotional stimuli, it is thought to represent similar underlying cognitive effects. However, it is differentiated from the EPN by a different topography and polarity, but it is not yet clear if this is because these are merely opposite ends of a dipole, or if there are distinct neural generators for the two components. Further research is needed to differentiate these two components. We have included a brief discussion of this, and all of the research we are aware of on the EAP or similar components in the aforementioned revisions addressing this concern.

2. We have elaborated on the potential implications of the LPP results in the discussion of experiment 2 (p. 25) and also expanded some relevant sections of the general discussion (p. 26, second paragraph) as requested by the reviewer. Additionally, see next.
3. The fact that words belong to different grammatical classes has been added as a limitation to the general discussion, and its possible impact on the LPP result is discussed (p. 30).
4. The time windows have been noted on the figures.

**Miniscule Issues**

1. We have double checked the in-text citations and made sure they are all correct.
2. The last sentence of the first paragraph on the page 12 has been edited to be more explicit (now the last line of the first paragraph on p. 14 of the All Markup changes manuscript).
3. The caption for table 4 has been updated as requested (p. 21).

**References**

Garcia, D., Garas, A. & Schweitzer, F. (2012). Positive words carry less information than negative words. *EPJ Data Science. 1*(3).

Grainger, J., Lopez, D., Eddy, M., Dufau, S., & Holcomb, P. J. (2012). How word frequency modulates masked repetition priming: An ERP investigation. *Psychophysiology, 49*(5), 604-616. <https://doi.org/10.1111/j.1469-8986.2011.01337.x>

Hauk, O., & Pulvermüller, F. (2004). Effects of word length and frequency on the human event-related potential. *Clinical Neurophysiology, 115*(5), 1090-1103. https://doi.org/10.1016/j.clinph.2003.12.020

Herbert, C., Kissler, J., Junghöfer, M., Peyk, P., & Rockstroh, B., (2006). Processing of emotional adjectives: Evidence from startle EMG and ERPs. *Psychophysiology, 43*, 197-206. doi: 10.1111/j.1469-8986.2006.00385.x.

Strijkers, K., Costa, A., & Thierry, G. (2010). Tracking lexical access in speech production: electrophysiological correlates of word frequency and cognate effects. *Cerebral cortex, 20*(4), 912-928. https://doi.org/10.1093/cercor/bhp153
