# Supplementary material for: The role of selective attention in the positivity offset: Evidence from event related potentials
Source: PLoS One. 2021 Nov 3;16(11):e0258640. doi: 10.1371/journal.pone.0258640 (PMC8565729; doi:10.1371/journal.pone.0258640)
Supplement: Attachment — Submitted filename: SelectiveAttentionPositivityOffset_PLOS_ResponsetoReviewer2.docx [file pone.0258640.s003.docx]

**Response to Reviewers**

Dear Dr. Hinojosa,

We would like to thank you and the reviewer for your consideration of our paper. We have made changes to the manuscript in response to the concerns noted. These are described below, along with additional explanations addressing each of their concerns.

We hope that these revisions, are well received. Changes in the revised manuscript are denoted in the file “SelectiveAttentionPositivityOffset_PLOS_TrackChanges2” with red font. Page numbers and paragraph information directing reviewers to the revisions noted below refer to the marked up manuscript. Thank you for your time and feedback. We look forward to your response.

Sincerely,

Regard Booy

**Major concerns:**

1. The point of concern noted by the reviewer was the unmatched word frequency values between negative, neutral and positive words. Specifically, that frequency for negative words was significantly lower than both positive and neutral words. We would like to stress that we agree with the reviewer on the importance of word frequency, and we thank them for their careful consideration of this issue.

The reviewer notes that we dismissed the possibility of word frequency effects on the later ERP components too readily and provides a number of studies showing the effect of low frequency on the N400, a component that temporally overlaps with the early part of our LPP window. It was not our intention to dismiss the importance of word frequency, but rather to emphasize the role emotional valence in the present study. We apologize that this was not evident and have revised the discussion of this issue accordingly. Specifically, as per the reviewer’s suggestion, in the discussion on p. 29 we note the possible influences of low frequency words on later ERP components and advise that the results be interpreted with caution.
